# Prediction of Ewing Sarcoma treatment outcome using attenuated tissue reflection FTIR tissue spectroscopy

**DOI:** 10.1038/s41598-018-29795-8

**Published:** 2018-08-17

**Authors:** Radosław Chaber, Kornelia Łach, Christopher J. Arthur, Anna Raciborska, Elżbieta Michalak, Krzysztof Ciebiera, Katarzyna Bilska, Katarzyna Drabko, Józef Cebulski

**Affiliations:** 1 Clinic of Paediatric Oncology and Haematology, Faculty of Medicine, University of Rzeszow, Rzeszow, Poland; 2 School of Chemistry, University of Bristol, Bristol, United Kingdom; 3 Department of Oncology and Surgical Oncology for Children and Youth, Institute of Mother and Child, Warsaw, Poland; 4 Department of Pathology, Institute of Mother and Child, Warsaw, Poland; 5 Institute of Informatics, University of Warsaw, Warsaw, Poland; 6 Department of Pediatric Hematology, Oncology and Bone Marrow Transplant, Medical University of Lublin, Lublin, Poland; 7 Center for Innovation and Transfer of Natural Sciences and Engineering Knowledge, University of Rzeszow, Rzeszow, Poland

## Abstract

Ewing sarcoma is the second most common type of primary bone cancer and predominantly affects children and young people. Improved outcome prediction is key to delivering risk-adjusted, appropriate and effective care to cancer patients. Advances in the Fourier Transform Infrared (FTIR) spectroscopy of tissues enable it to be a non-invasive method to obtain information about the biochemical content of any biological sample. In this retrospective study, attenuated tissue reflection FTIR spectroscopy of biopsy samples from paediatric patients reveals spectral features that are diagnostic for Ewing Sarcoma. Furthermore, our results suggest that spectral features such as these may be of value for the prediction of treatment outcome independent to well-known, routinely used risk factors.

## Introduction

Ewing sarcoma (ES) is a devastating, poorly differentiated, mesenchymal tumour of bones or soft tissues. It is the second most common type of primary bone cancer in the US and Europe where it accounts for approximately 25% to 34% of malignant bone tumours^1^. ES is rare, affecting about 2.9 people per million annually, however, predominantly affects children and young adults under 20 years of age^2^. This disease accounts for 2% of childhood cancers^3^.

With advances in multimodal therapy, survival rates for patients with localized disease approach 75%^4,5^. In contrast, patients with metastatic, refractory, or relapsed disease have very poor outcomes generally^6–8^. The 5-year survival rate for patients with metastases detected at diagnosis remains around 25% and even around 10% when relapse occurs within the first 2 years following treatment^9^. International studies conducted over the last three decades have identified clinically relevant prognostic factors, and clinical characteristics such as age, tumour size and site, metastatic pattern and histologic response to the neoadjuvant chemotherapy measured by the percentage of viable tumour cells remaining after its completion. Consequently, 18F-FDG PET/CT results are now being investigated for future risk-adapted therapies by various groups^4,5,10^. There remains an unmet need for improved prognostic methods which can enable more accurate and effective application of such risk-adapted therapies.

Fourier Transform Infrared (FTIR) Spectroscopy is a non-invasive physicochemical method which measures the absorption of infrared radiation from chemical bonds in functional groups of molecules. It is one of the most relevant tools to characterize *ex vivo* tissues because this technique provides bulk information about the biochemical content of a biological sample in contrast with immunohistochemistry or other molecular techniques that target one macromolecule type at a time^11^. The frequency range of absorption by molecules is correlated with their structure^12^. It can be applied to study among others the nucleic acid strands (1000–1250 cm^−1^), proteins (1500–1560 cm^−1^, 1600–1700 cm^−1^), lipids (2800–3000 cm^−1^) as well as inorganic structures as phosphates (460–1120 cm^−1^)^13^. FTIR has many advantages for analyzing of biological structures like the small sample size (a few micrograms) required for analysis, no need for pre-processing of tissue (*e.g*., demineralization, staining), no need for tissue dewaxing^14,15^ and does not require the use of biological markers^16^. Due to these advantages, FTIR can be applied to the very early stages of the disease, before the presence of changes is detectable by light microscopy^17^, and may be suitable for the monitoring a disease course and therapeutic outcome^13^. They may be useful in oncologic diagnostics^13,18–20^ but also for the comparison of spectra obtained before and after cancer chemotherapy^11,17,21–23^. Data analysis methods allow spectrum transformation, baseline correction, normalization and smoothing enable the quantitative analysis of FTIR spectra and have been applied to biology and medicine^13^.

This retrospective study is based on primary ES bone tissue sections from 27 pediatric patients and demonstrates that FTIR spectroscopy may be a potential prognostic factor of relapse/progression-free survival and overall survival before chemotherapy administration.

## Material and Methods

### Patients

Twenty-seven patients (age 5–20 years old) with newly diagnosed Ewing sarcoma of bone were included in this study. Each patient was treated according to the Euro-EWING protocols in the years 2010–2016 and their clinical characteristics are presented in Table 1

**Table 1:** The clinical characteristic of patients (n = 27).

**gender** male/female	12/15 pts.
**age** [yrs] range median <10 yrs old >=10 yrs old	5–20 yrs 14 yrs 9 pts 18 pts
**localized** **metastases** - lungs - bones - others	11 16 14 5 1
**tumor microscopically** **incompletely resected**	1
**histological response** **to first line CTx (good/poor)**	20/7
**local radiotherapy** **auto HSCT**	17 9
**relapses(progressions)/deaths**	14/9
**follow up time [months]** range median	14–74 29

. In each case, identical induction neoadjuvant chemotherapy (neo-CTx) of six VIDE cycles (vincristine, ifosfamide, doxorubicin, etoposide) were administrated. Surgery was performed in every patient at the Department of Surgical Oncology, Institute of Mother and Child in Warsaw, Poland. Microscopically complete resection was possible in the 25 cases. Each histopathological sample was verified centrally at the same institution as well as their assessment of response to chemotherapy measured by the percentage of viable tumour cells remaining after neo-CTx completion. Good response was defined as greater than or equal to 90% necrosis, and a poor response was defined as less than 90% of necrosis. Postoperative treatment was dependent on the individual clinical status of the patient and was also conducted according to Euro-EWING protocols. There were no deaths observed for reasons other than cancer progression. Informed consent was obtained from all patients or their guardians before treatment. This retrospective study was conducted under Institutional Review Board (Protocol No. KBET/6/06/2014) from June 2014 at University of Rzeszow. The experimental protocols used in this study were approved by the institutional ethics committees (IECs) of the University of Rzeszow, and were carried out in accordance with the approved guidelines.

### Samples preparation

Each patient’s samples included 3 types of formalin-fixed paraffin-embedded (FFPE) tissues which were analyzed by FTIR spectroscopy. The first FFPE blocks consisted of ES tissues collected during a diagnostic biopsy performed before ES therapy. The second group of FFPE blocks consisted of tumour tissue after 6 courses of neo-CTX. The third group of FFPE blocks consisted of normal bone tissue from the patients, obtained outside the area of the ES infiltration (verified microscopically). All samples were prepared and verified by pathologists experienced in ES.

Histological blocks of bone tissue, embedded in paraffin, were sectioned to a thickness of 10 µm using a rotary microtome. FFPE tissue sections were then applied to calcium fluoride slides. In the first phase of labelling, sections were placed on the surface of a tub filled with hot water to allow them to smooth. At this point, the sample was gently pulled onto a slide.

Samples were then dewaxed by washing twice in xylene. Samples were rehydrated by rinsing in an alcohol series from absolute alcohol (99.8%) through 96%, 80%, and 70% alcohol. Finally, samples were rinsed with distilled water and dried. Each incubation step lasted for 5 minutes.

### FTIR spectroscopy

FTIR spectra were recorded using a Bruker Vertex 70 v Fourier transform infrared spectrometer (FT-IR). Tissue specimens were applied to the attenuated total reflection (ATR) plate. Mid-infrared (IR) radiation was passed through the sample using a single-reflection snap ATR crystal diamond. To achieve 2 cm^−1^ of spectral resolution 32 scans were conducted. Spectra were recorded in the range of 800–3500 cm^−1^. As the samples were dewaxed, the air was measured as the background. All measurements were recorded in triplicate. Initial data analysis was performed using the program OPUS 7.0 from Bruker Optik GmbH 2011. Spectra were normalized, and baseline corrections were made.

Our previous preliminary study^24^, had revealed that wave-numbers in the range 950–1100 cm^−1^ are the most relevant for monitoring ES clinical course. The wave-number corresponding to the position of absorbance peak maximum in this range was subsequently analyzed and compared with clinical data. Three measurements were obtained: from ES tumour sample (T_b_) and from the normal bone tissue (T_n_) outside the tumour area from the same biopsy sample. The third one was taken from the tumour resected after the sixth VIDE chemotherapy cycle (T_t_).

### Statistical analysis

The spectral data were analyzed by a two-way ANOVA followed by Tukey’s test to determine the correlation between the functional groups of chemical compounds. Correlation between wavenumber and histologic evaluation of percent necrosis were analyzed using the two-tailed Pearson correlation coefficient. Overall survival was defined as the time interval from the date of diagnosis to the date of death from any cause or to last follow-up date. Progression-free survival (PFS) was taken to be the time from diagnosis until disease progression, relapse or last patient contact. The overall survival and progression-free survival curves were estimated according to the Kaplan–Meier method and compared using the log-rank test. The parameters evaluated in univariate analysis were used as explanatory variables in Cox regression models of progression-free survival in the final multivariate models. In the multivariate analyses, only factors identified as significant by the univariate analysis were investigated.

The clinical groups were compared by using the Mann–Whitney U test. The optimal wave number cut-off point to distinguish a patient with the highest probability of unfavourable outcome (disease progression/relapse/death) was determined using Receiver Operating Characteristic [ROC] analysis implementing the Youden index. It was assumed that the cost of obtaining a false negative is twice as high as the cost of obtaining a false positive. A 2-sided *P* value of <0.05 was defined as statistically significant. The calculations were performed using Statistica 12; StatSoft. 2016. Medical kit version 3.0.

## Results

**Figure 1:**
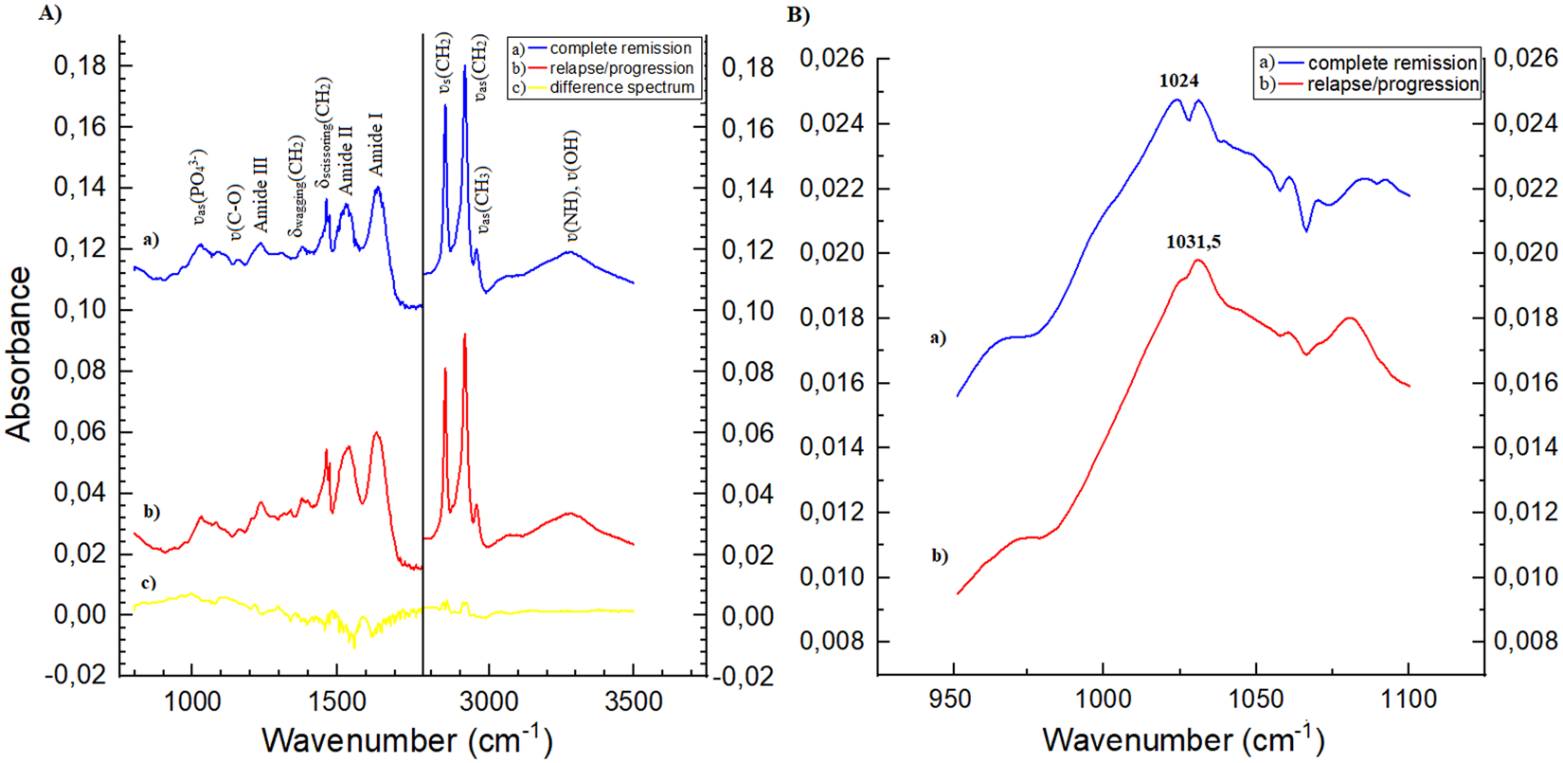
(**A**) Mean spectra of ES primary tumors sampled at diagnosis (T_b_) of patients with complete remission (a) and with relapse/progression during 3 years from diagnosis (b) with assignment of bands to the main chemicals groups composing macromolecules. The symbols νs, νas, δs stand for symmetric stretching vibrations, asymmetric stretching vibrations, and symmetric bending vibrations, respectively. The difference spectrum (**c**) is obtained by subtracting spectra of patients with relapse/progression from spectra of patients in complete remission. Measuring range 800–1800 and 2800–3500 cm^−1^. (**B**) Mean spectra FTIR of the maximum absorbance peak from 950–1100 cm^−1^ range in ES tumors at diagnosis for: patients with complete remission (a) and with relapse/progression during 3 years (b) from diagnosis. Measuring range 900–1200 cm^−1^.

Mean ATR-FTIR spectra of ES primary tumours sampled at diagnosis (T_b_) of patients with complete remission and with relapse/progression during 3 years from diagnosis are presented in Fig. 1. The median wavenumber of maximum absorbance peak from 950–1100 cm^−1^ FTIR spectrum from ES tumour biopsy before neo-CTx (T_b_) was 1030 cm^−1^ for the whole analyzed group and it is significantly lower than the median for normal bone tissue (T_n_) – 1036.5 cm^−1^ (p = 0.04). The median wavenumber for resected ES tumour after neo-CTx completion (T_t_) was 1031 cm^−1^ but this is not statistically significant to both T_b_ and T_n_.

The obtained results (the medians with range) for T_b_, T_n_ and T_t_ wave-number values, as well as the shifts of the peaks expressed as differences T_b_ − T_n_ and T_t_ − T_b_, are presented in Table 2. No important differences were observed among patient subgroups distinguished according to clinical features with exception of significant different values of T_b_ − T_n_ and T_t_ − T_b_ in patients younger and older than 15 years old and T_t_ − T_b_ between boys and girls. The treatment modalities, like autologous stem cell transplantation or radiotherapy as well as VAC (vincristine, dactinomycin, cyclophosphamide) versus VAI (vincristine, dactinomycin, ifosfamide) adjuvant chemotherapy cycles had no significant impact on the outcome. There were observed no correlations between necrosis after neo-CTx completion percentage and respectively: T_b_ (R Spearman = −0.211; p = 0.29), T_t_ (R Spearman = 0.08; p = 0.67), T_n_ (R Spearman = −0.213; p = 0.37), T_b_ − T_n_ (R Spearman = −0.11; p = 0.64) and T_t_ − T_b_ (R Spearman = 0.27; p = 0.17).

**Table 2:** The wavenumber median of maximum absorbance peak from 900–1100 cm^−1^ FTIR spectrum from ES tumor before (T_b_), after (T_t_) neo-CTx completion and normal bone tissue (T_n_) at diagnostic biopsy.

Variable (n=)		T_b_ [cm^−1^] median (range)	p	T_t_ [cm^−1^] median (range)	p	T_n_^*^ [cm^−1^] median (range)	p	T_t_ − T_b_ [cm^−1^] median (range)	p	T_b_ − T_n_^*^ [cm^−1^] median (range)	p
gender	male (12)	1026.0 (1019–1031)	0.37	1031.0 (1018–1080)	0.68	1040 (1029–1080)	0.27	−2,0 (−24–+29)	**0.05**	−0,5 (−26–+27)	0.24
	female (15)	1030.5 (989–1081)		1031.0 (1023–1082)		1036 (1014–1080)		+5.0 (−3–+33)		−8.0 (−19–+18)	
age	<15 yrs old (17)	1026.0 (989–1079)	0.14	1031.0 (1018–1072)	0.94	1041.0 (1027–1080)	0.94	+6 (−7–+33)	**0.008**	−9.0 (−19–1)	**0.024**
	≥15 yrs old (10)	1031.5 (1024–1081)		1031.0 (1020–1077)		1031.0 (1014–1079)		−2.5 (−24–+7)		+2.0 (−26–+27)	
disease	local (11)	1030.0 (989–1061)	0.79	1031.0 (1018–1082)	0.15	1031.0 (1014–1080)	0.76	+4.0 (−7–+29)	0.61	−3.0 (−26–+18)	0.82
	metastatic (16)	1028.0 (1019–1081)		1032.0 (1023–1080)		1044.0 (1027–1080)		+5.5 (−24–+33)		−7.0 (−19–+27)	
site of tumor	peripheral (20)	1028.5 (989–1079)	0.57	1031.0 (1018–1080)	0.06	1036.5 (1014–1080)	0.60	+3.0 (−24–+29)	0.20	−6.5 (−26–+27)	0.78
	axial/pelvis (7)	1030.0 (1020–1081)		1050.0 (1030–1082)		1039.5 (1029–1080)		+5.0 (−4–+33)		−6.0 (−19–+2)	
necrosis	favorable (≥90%) (20)	1026.5 (1019–1079)	0.93	1031.0 (1023–1082)	0.13	1036.5 (1014–1080)	0.44	+5.5 (−24–+33)	0.08	−7.0 (−19–+27)	0.62
	unfavorable (<90%) (7)	1030.0 (989–1081)		1024.0 (1018–1077)		1040.5 (1031–1079)		−4.0 (−7–+29)		−0.5 (−26–+2)	
relapse/progression	yes (14)	1031.5 (1025–1081)	**0,0007**	1031.5 (1023–1080)	0.37	1031.5 (1014–1080)	0.73	−0.5 (−24–+14)	**0.0022**	−1.0 (−14–+27)	**0.0015**
	no (13)	1024.0 (989–1061)		1031.0 (1018–1082)		1044.5 (1029–1080)		+7.0 (−4–+33)		−16.0 (−26–3)	
death	yes (9)	1033.0 (1030–1081)	**0.0012**	1034.0 (1024–1080)	**0.035**	1039.0 (1029–1080)	0.43	0.0 (−24–+20)	0.076	−0.5 (−18–+27)	0.058
	no (18)	1025.0 (989–1061)		1031.0 (1018–1082)		1036.5 (1014–1080)		+5.5 (−6–+33)		−8.0 (−26–+18)	
total (27)		1030.0 (989–1081)	—	1031.0 (1018–1082)	—	1036.5 (1014–1080)	—	+4.0 (−24–+33)	—	−6.5 (−26–+27)	—

The wavenumbers were compared using U Mann-Whitney test. Significant differences are indicated in bold.

^*^The measurement of FTIR spectrum for normal bone tissue was possible to obtain in 20 cases.

Unexpectedly, very significant differences in T_b_ values were observed between patients with relapse/disease progression and those being in complete remission. A similar significant difference was visible in the median T_b_ values between children who died and survivors. Parameters like T_b_ − T_n_ and T_t_ − T_b_ were also significantly different in patients with relapsed/progression compared to the survivors.

Receiver operating characteristic curves were used to establish the most suitable values for T_b,_ T_b_ − T_n_ and T_t_ − T_b_ cut-off which could separate the group of patients with poor prognosis (with ES progression/relapse or death). According to this Analysis we established the following cut-off values: T_b_ ≥ 1027 cm^−1^, T_t_ − T_b_ < + 1 cm^−1^ (what is equal with the shift of the maximum peak wavenumber after neo-CTx to the left or up to 1 cm^−1^ to the right) and T_b_ − T_n_ ≥ −1 cm^−1^ (the shift of the normal bone tissue peak to the right or up to 1 cm ^−1^ to the left of the T_b_ position). The area under curve (AUC) were respectively: 0.865; 0.832 and 0.901.

**Figure 2:**
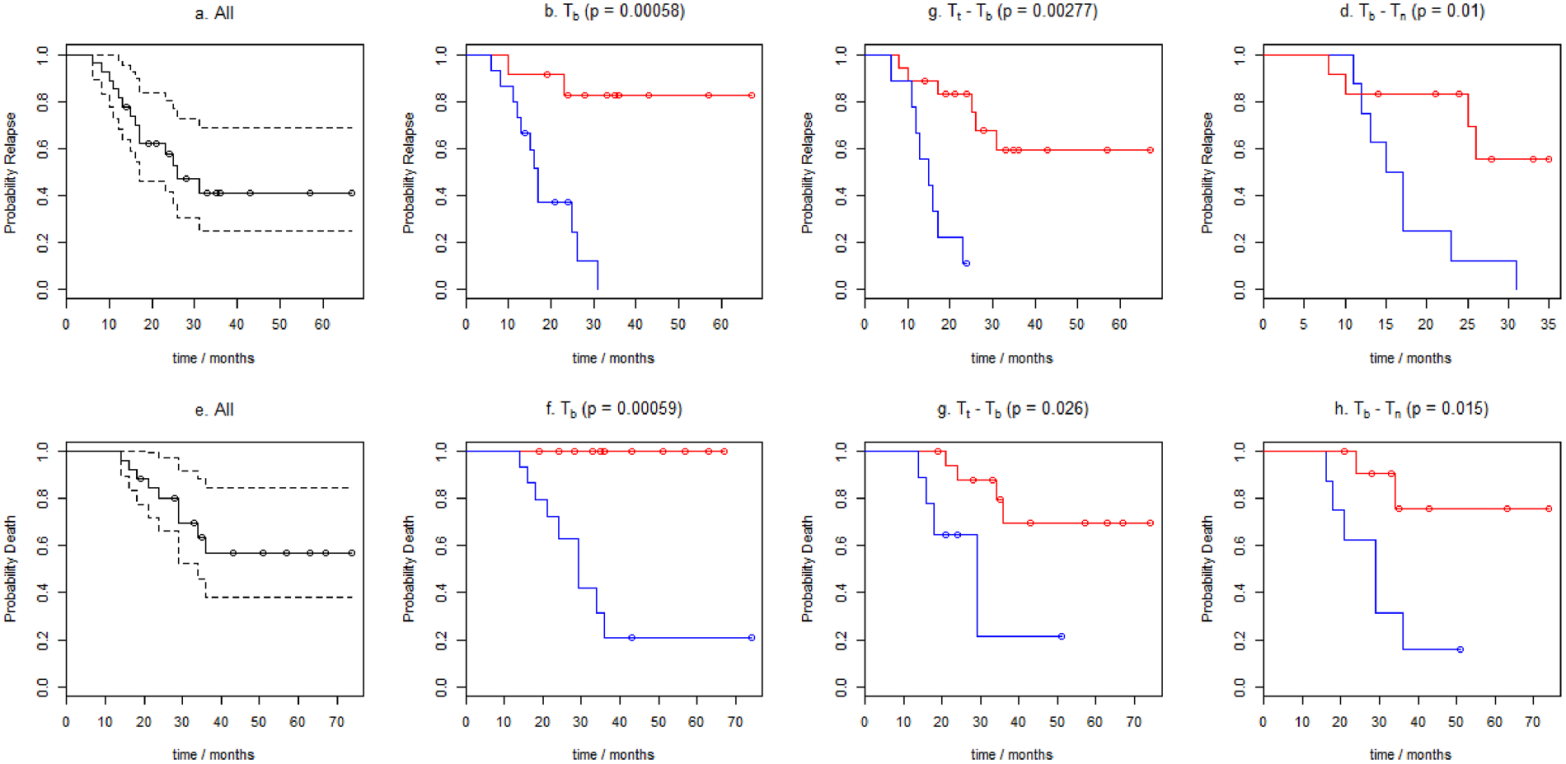
Kaplan–Meier curves comparing progression-free survival (PFS) (**a**–**d**) and overall survival (OS) (**e**–**h**) in patients according to the wave-number corresponding to the position of absorbance peak maximum in the range 950–1100 cm^−1^:  from Ewing Sarcoma tumour sample (T_b_) and the shifts of the peaks expressed as differences T_b_ − T_n_ (T_n_ – the wave-number from the normal bone tissue); T_t_ − T_b_ (T_t_  - the wave-number from tumour resected after 6^th^ VIDE chemotherapy cycle). (**b**) and **f**) T_b_ ≥ 1027 cm^−1^ (Blue) vs < 1027 cm^−1^ (Red); (**c**) and **g**) T_t_ − T_b_ ≥ +1 cm^−1^ (Red) vs T_t_ − T_b_ < +1 cm^−1^ (Blue) (**d**) and (**h**) T_b_ − T_n_ < −1 cm^−1^ (Red) vs T_b_ − T_n_ > = −1 cm^−1^ (Blue).

The median follow-up for the analyzed patients was 29 months (14–74 months). The 3-year progression-free survival rate was 41,36%, and the 3-year overall survival (OS) rate was 56,66%. Considering only well-known, classical risk factors in ES^25^ (presented in Table 2), only patient gender had a significant impact on 3 yrs survival: Overall survival (p = 0.01) in the univariate analysis and nearly significant on progression-free survival at 3 yrs (p = 0,06). Across the whole patient group, the median T_b_ value and differences T_b_ − T_n_ and T_t_ − T_b_ were found to be strongly related to outcome (Table 3), e.g. 100% of patients with T_b_ ≥ 1027 cm^−1^ (n = 15) had progressed or relapsed during the 3 yrs follow up period compared to 82% progression-free survival in patients with T_b_ < 1027 cm^−1^ in this period (n = 12; log-rank p = 0.00058). Kaplan-Meier plots for progression-free survival and overall survival according to variables established in ROC analysis: T_b_, T_b_ − T_n_ and T_t_ − T_b_ values are presented in Fig. 2

**Table 3:** Prognostic values of classical and spectral parameters on univariate analysis.

Variable	n	PFS 3yrs	p	OS 3yrs	p
**site of tumor** peripheral axial/pelvis	20 7	0.33 0.69	0.20	0.58 0.53	0.59
**age** < 15 >= 15 yrs old	17 10	0.50 0.27	0.19	0.59 0.47	0.47
**gender** male female	12 15	0.31 0.52	**0.05**	0.32 0.73	**0.01**
**disease** local metastatic	11 16	0.40 0.45	0.92	0.58 0.54	0.65
**necrosis** favorable (≥90%) unfavorable (<90%)	20 7	0.50 0.21	0.31	0.67 0.23	0.1
**radiotherapy RTx** yes no	17 10	0.35 0.55	0.25	0.59 0.87	0.07
**autoHSCT** yes no	8 19	0.16 0.55	0.23	0.44 0.80	0.05
**adjuvant CTx** VAI VAC	18 6	0.43 0.41	0.57	0.71 0.45	0.16
**T**_**b**_**≥1027** [cm^−1^] **T**_**b**_**< 1027** [cm^−1^]	15 12	0.00 0.82	**0.00058**	0.21 1.00	**0.00059**
**T**_**t**_**-T**_**b**_ ≥ +1 [cm^−1^] **T**_**t**_**-T**_**b**_ < +1 [cm^−1^]	18 9	0.65 0.00	**0.00277**	0.69 0.21	**0.026**
**T**_**b**_**-T**_**n**_ ≥ −1 [cm^−1^]* T_**b**_**-T**_**n**_ < −1 [cm^−1^]*	8 12	0.00 0.55	**0.01**	0.30 0.73	**0.015**

Significant differences are indicated in bold.

*The measurement of FTIR spectrum for normal bone tissue was possible to obtain in 20 cases.

.

In the multivariate analysis for percentage progression-free survival (Table 4), T_b_ values of 1027 cm^−1^ or more remains the most significant independent prognostic factor (p = 0.004; HR = 10.41; 95% CI 2.07–52.2), and T_t_ − T_b_ < +1 cm^−1^ emerged as the second most important independent variable (*p*

**Table 4:** Multivariate Cox Regression Analysis for PFS with Backward Wald Statistics Method (27 Patients).

Parameter	Risk of relapse/progression p=0,00005			
	Wald test (p)	HR	95% C.I. HR	
male gender	0.69	1,37	0.29	6.55
**T**_**b**_ **≥ 1027 [cm** ^− **1**^ **]**	**0.004**	**10.41**	**2.07**	**52.20**
**T**_**t**_ **-T**_**b**_ **< +1 [cm** ^− **1**^ **]**	**0.034**	**6.57**	**1.15**	**37.54**
**T**_**b**_-**T**_**n**_ ≥−1 [cm^−1^]*	0.74	1.33	0.24	7.53

Significant results are indicated in bold. *T_b_-T_n_ measurements in 20 cases (p = 0,014).

= 0.034; HR = 6.57; 95% CI 1.15–37.54). The p-value of male gender was not significant in this analysis.

## Discussion

ATR-FTIR spectroscopy can reveal structural and compositional changes of cells at the molecular level. These changes, arising from carcinogenesis or the action of cytotoxic drugs, cause alteration of the levels of biomolecules within the cells. Due to the different vibrational modes of these biomolecules, a change in the bulk vibrational spectra of the tissue occurs. Chemometric statistical methods can allow this spectral change data to be used for exploratory and modelling purposes allowing normal or malignant cells to be differentiated based on their spectral characteristics^18^.

The analysis of bone samples can be costly and time-consuming. In contrast, some significant advantages of this method are its speed and low-cost. We have shown previously^15^ that paraffin dewaxing can be avoided in the sample preparation procedure which decreases the risk of bone material damage facilitating the use of the method in routine and scientific analyses.

To date, there have been no published attempts to apply any vibrational spectroscopic method (such as infrared, near-infrared and Raman) to the study of Ewing Sarcoma for progression modelling. Wald *et al*.^11^, have demonstrated that unsupervised analysis of the FTIR spectra evidence permitted the assignment of patients with primary melanoma into two groups that correlated to the clinical responsiveness of the patients to dacarbazine used as a first-line treatment. A supervised model resulted in correct identification of patient status (responder/non-responder) in 83% of cases. Furthermore, the spectra revealed a key modification in the nature and quantity of lipids in the cells of both groups. Zawlik *et al*. have reported the application of a focal-plane-array Fourier transform infrared (FPA-FTIR) spectroscopy combined with principal component analysis (PCA) that allowed the effect of chemotherapy on triple-negative breast cancer patients to be monitored^21^. The PCA results obtained using the FPA-FTIR spectral data collected before and after the chemotherapy revealed discriminatory features that were consistent with the pathologic and clinical responses to chemotherapy, indicating the potential of the technique as a monitoring tool for observing chemotherapy efficacy. Tolstorozhev *et al*. have found that infrared spectroscopic analysis can be used in the diagnosis and treatment monitoring of cancers of various organs at the molecular level^22^. They found that when lung malignancy was treated with palladium complexes of methylenediphosphonic acid, the spectroscopic signs of the presence of metastases in the lungs disappear, and the infrared spectrum of the lung tissue after treatment practically coincides with the spectrum of healthy lung tissue. Significant changes in cellular lipid composition were observed by Denbigh *et al*. upon treatment of K562 and HL60 cells (acute myeloid leukaemia) with the drug combination of bezafibrate and medroxyprogesterone acetate when examined by ATR-FTIR, Synchrotron radiation FTIR (S-FTIR) and Raman micro-spectroscopy^23^. The emerging diagnostic applications of FTIR spectroscopy are growing as the field matures and it has been applied successfully to the study of breast cancer^17^, lung cancer^26^, ovarian cancer^27^, brain tumors^28^, cervical cancer^29^, gastric cancer^30^, colon cancer^31^, prostate cancer^32^. The preliminary results of FTIR spectroscopy application in diagnostics of a bone tumour have been reported in our previous studies^15,24^ emphasizing the applicability of FTIR to all tissue types.

The “bio-fingerprint region” (1800 cm^−1^ to 900 cm^−1^) of the mid-IR spectrum contains the fundamental vibrational modes of key chemical bonds that may be exploited to provide a rapid non-destructive, screening approach to diagnosis^33^. We, therefore, sought to identify specific spectral features in ES tissue as the equivalent of a traditional cancer biomarker. We showed the strong predictive value of the wavenumber of the maximum peak absorbance in the range of 1100–900 cm^−1^ in ES samples at diagnosis before neo-CTX administration (T_b_), as well as the wave numbers difference between this peak position and its equivalents in the resected tumor sample after neo-CTx completion (T_t_ − T_b_) or from the normal bone tissue sample (T_b_ − T_n_) taken from the same patient. Unexpectedly, in our small cohort of patients these features are the strongest independent factors for outcome prognosis (PFS) in the analyzed group. The progression-free survival and overall-survival times of these patients could be predicted more accurately using these FTIR spectral parameters than from the standard, clinical prognostic factors like presence of distal metastases at diagnosis, the primary tumor localization (axial *vs* peripheral) and the histological response (greater than or equal to 90% necrosis after neo-CTx). From the clinical features, only gender was significantly predictive for prognosis in the univariate and multivariate analyses. We believe that the small size of our analyzed patient group limits the prognostic superiority of this spectral parameter over routinely used risk factors.

In our previous, preliminary study^24^ we postulated that the shift of the maximum absorbance peak in the range 1100 cm^−1^–900 cm^−1^, which was related to chemotherapy response, is dependent on signals derived from the phosphate group in the sample. Presently, based on the greater results of the spectral analysis presented herein, we have to revise this conclusion and state that it is impossible to unequivocally attribute this peak only to one chemical structure as the vibrations of different molecular components of a cell overlap and the spectrum thus reflects the average biochemical composition^13^. Conclusive biological explanation of the tissue-section spectral results is impossible without inviting over-analysis. Due to this limitation, the addition of the other analytical instruments like electron paramagnetic resonance spectroscopy, nuclear magnetic resonance spectroscopy, Raman spectroscopy or X-ray absorption near edge structure is necessary for a better understanding of phenomena connected with ES aetiology and clinical course, occurring in the tumour cells. Presently, we can only speculate that the maximum absorbance peak responsible for outcome prediction in ES can be associated to the biochemical components containing phosphates like nucleotides from DNA or RNA, phospholipids from cell membranes, phosphorylated proteins, phosphate esters but may also arise from hydroxyapatite which is the fundamental bone component^13,34,35^. Likewise, ES cells contain glycogen particles with corresponding FTIR spectroscopy wave-numbers from 1000–1100 cm^−1 ^^13,26^ and it is thus likely that glycogen contributes to the final shape and position of this peak.

## Conclusions

The identification of clinically applicable methods that allow the adoption of risk-adapted therapeutic approaches is an unmet need in the treatment of bone cancers such as Ewing Sarcoma. In this retrospective study, we demonstrate that selected spectral FTIR parameters related with wave-numbers corresponding to maximum peak absorbance from 950–1100 cm^−1^ range may have a strong predictive value on the outcome (progression free survival, overall survival) of patients with Ewing sarcoma of bones treated according to VIDE and VAI/VAC based chemotherapy regimens.

Significantly two of the three described spectral parameters (T_b_ and T_b_ − T_n_) can be obtained prior to the the start of the treatment. It may, therefore, be possible to assess the likelihood of treatment success, thus allowing alternate treatment regiments to be undertaken in patients whose predicted response to VIDE and VAI/VAC based chemotherapy is poor. Moreover, as ES is rare and it presents with symptoms similar to other conditions patients are frequently misdiagnosed or patient diagnosis is delayed. In addition, although the conventional immunohistochemical marker for Ewing Sarcoma (CD99) has high sensitivity it is also low specificity for the disease. We therefore feel that FTIR may offer an additional, complementary tool for the improvement of ES diagnostics.

Although it is not possible from this study to determine the potential levels of specificity and sensitivity when using FTIR to discriminate prognostic outcome our results suggest that it is worthy of further research. We therefore hope to investigate this further on more numerous and diverse patient groups in the hope that these spectral features may be strongly predictive for the identification of individuals with the worst prognosis.

### Compliance with ethical standards

All procedures performed in studies involving human participants were in accordance with the ethical standards of the institutional and/or national research committee and with the 1964 Helsinki declaration and its later amendments or comparable ethical standards. Informed consent was obtained from all individual participants included in the study.

## Electronic supplementary material

Dataset 1
